# Microbial metabolites short chain fatty acids, tight junction, gap junction, and reproduction: a review

**DOI:** 10.3389/fcell.2025.1624415

**Published:** 2025-08-22

**Authors:** Lulu Fu, Min Wang, Dan Li, Shuai Ma, Fuliang Zhang, Lianwen Zheng

**Affiliations:** ^1^ Reproductive Medical Center, The Second Hospital of Jilin University, Changchun, China; ^2^ Stroke center,Department of Neurology, The First Hospital of Jilin University, Changchun, China

**Keywords:** gut microbiota, short chain fatty acids, tight junction, gap junction, reproduction

## Abstract

The gut microbiota, comprising trillions of bacteria, fungi, and viruses, exists in symbiosis with the host. As the largest microbial ecosystem in the human body. The gut microbiota not only shapes the homeostasis of the intestinal microenvironment through gut-derived metabolites but also exerts regulatory effects on the functions of diverse tissues and organs throughout the body via the intricate “gut-distal organ axis” mechanism. Short chain fatty acids, such as acetic acid, propionic acid and butyric acid are high abundance intestinal metabolites, not only influence the intestinal barrier by regulating tight junction proteins, but also affect intestinal peristalsis by regulating gap junction proteins. These microbial metabolites may also play a important role in the formation and maintenance of the key barriers of the reproductive system, such as the ovarian blood follicle barrier, the testicular blood-testis barrier, and the endometrial epithelial barrier. In reproductive system, Gap junction-mediated intercellular communication, facilitated by connexins, proves essential in germ cell maturation, embryo implantation, and spermatogenesis. The dysregulation of these microbial metabolites leading to abnormal tight junction and gap junction protein functions provides novel perspectives for understanding the pathogenesis of reproductive disorders such as polycystic ovary syndrome and premature ovarian failure. This review systematically elucidates the molecular networks through which short-chain fatty acids regulate tight and gap junction proteins, highlighting their potential roles in reproductive physiology.

## 1 Introduction

The gut surface is the largest surface of the human body, covering about 200–300 square meters (Dommett et al., 2005). There are more than 100 trillion microorganisms of various types on the gut surface, including bacteria, fungi, and viruses, which constitute a complex and dynamic ecosystem within the gut (Abdul Rahim et al., 2019). The gut microbiota-derived metabolites can regulate various metabolic pathways through receptor-activated cascade signaling on gut cell surfaces and affect gut health, and can also regulate the physiological functions of other organs and tissues through blood circulation (Abdul Rahim et al., 2019). Therefore, the imbalance of gut microbiota may lead to the occurrence of a variety of diseases.

**FIGURE 1:**
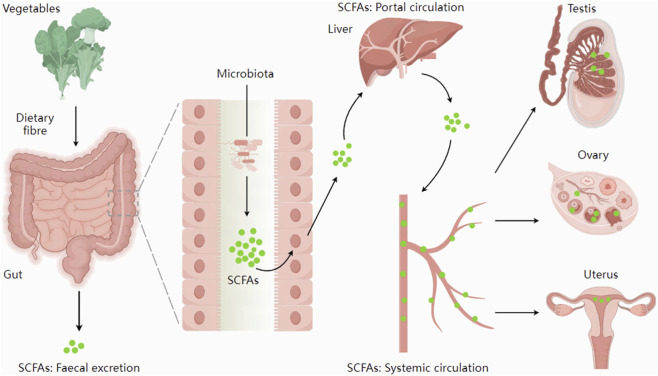
The synthesis and distribution of SCFAs. Short-chain fatty acids, synthesized by the gut microbiota, are absorbed through the intestines and enter the systemic circulation via the portal vein. They could be transported through the bloodstream to reach the reproductive organs. Figure 1 was adapted from: Dalile et al. (2019).

The gut microbiota can produce a variety of metabolites. Studies have shown that the gut microbiota contains a wide variety of enzymes that catalyze biochemical reactions that cannot occur in host cells (Louis et al., 2014). In recent years, gut microbial metabolites—including short-chain fatty acids, phenolic acids, tryptophan, secondary bile acids, and other gut microbiota metabolites—have attracted increasing attention (Rothhammer et al., 2016). Short-chain fatty acids can be produced through the fermentation of resistant starch, proteins, and their digestive products (primarily peptides and amino acids) by the gut microbiota in the host’s cecum and colon (Deleu et al., 2021). These metabolites influence host health by participating in the maintenance of glucose homeostasis, immune responses, and intestinal barrier function (Chambers et al., 2018). Short-chain fatty acids can also be absorbed by gut cells as a source of energy, and unconsumed short-chain fatty acids circulate through the bloodstream to other tissues and organs (see Figure 1) (Dalile et al., 2019).

Tight junctions and gap junctions play important roles in maintaining intestinal health. Gap junctions are important for intercellular communication: they act as channels that allow ions and small molecules to spread between adjacent cells (Harris, 2001), on the other hand, tight junctions primarily form a permeability barrier, enabling selective permeability of cellular layers (Zihni et al., 2016). In the gut, gap junction connexin-43 (CX43) is involved in maintaining gut motility; reduced CX43 expression can lead to constipation (McClain et al., 2014; Gao et al., 2022). Additionally, decreased expression of CLDN11, a key tight-junction protein constituting the gut barrier (Li et al., 2021), leads to increased gut permeability (Lin et al., 2025). However, tight junctions and gap junctions are not limited to the gut; they are expressed in tissues and organs throughout the body. In the ovary, the loss of the key gap junction protein CX43 can lead to the arrest of female follicle growth (Winterhager and Kidder, 2015). In the uterus, alterations in the expression of the tight junction proteins CLDN3 and CLDN4 are associated with decreased implantation rates (Martínez-Peña et al., 2017). In the testis, CLDN11 also participates in the formation of the blood-testis barrier (Smith and Braun, 2012), and its reduced expression disrupts this barrier, causing spermatogenesis disturbances (Kanatsu-Shinohara et al., 2020). This evidence suggests that in pathological conditions characterized by intestinal barrier disruption or impaired gut motility, the core mechanism may parallel the dysfunction causing reproductive disorders. Central to these processes are claudin family proteins (key constituents of tight junctions governing barrier integrity) and connexin family proteins (essential for gap junction-mediated intercellular communication and motility regulation), both ubiquitously expressed across multiple organ systems.

Herein, we examined the effects of short-chain fatty acids on the gut barrier and motility, analyzed the roles of claudins and connexins in reproductive organs, and summarized research on short-chain fatty acids in the reproductive system. This led us to recognize potential connections between gut health and reproductive health.

## 2 Synthesis and distribution of short-chain fatty acids

Short-chain fatty acids (SCFAs) are aliphatic carboxylic acids with aliphatic tails containing five or fewer carbons, including formic acid (C1), acetic acid (C2), propionic acid (C3), butyric acid (C4), and valeric acid (C5). Among these, acetic acid, propionic acid, and butyric acid are the most abundant SCFAs in the gut, collectively accounting for over 95% of the total gut SCFAs, with their relative abundances in a ratio of approximately 3:1:1 (Macia et al., 2012). SCFAs are produced by gut bacteria via fermentation of cellulose and resistant starch, which cannot be directly metabolized and absorbed by the host in the small intestine (Hu et al., 2022). Acetate is synthesized through either the acetyl-CoA pathway or the Wood-Ljungdahl pathway (Ragsdale and Pierce, 2008). Propionate is primarily generated via the succinate pathway in *Bacteroides* and the lactate pathway in Firmicutes (Louis et al., 2014), while butyrate is synthesized from acetyl-CoA, butyryl-CoA, acetate, and lactate (Yang et al., 2021).

SCFAs exhibit distinct regional distributions in the gut: acetate and propionate are present in both the small and large intestines, whereas butyrate is predominantly localized to the colon and cecum (Vogt et al., 2015). The total SCFA concentration in the proximal colon is estimated to be 70–140 mM, decreasing to 20–70 mM in the distal colon (Bergman, 1990), indicating substantial colonic absorption of SCFAs as an energy source. A small fraction of unabsorbed SCFAs is excreted in stool, while the majority enters the hepatic portal vein. The portal venous blood SCFA concentration (375 μmol/L) is approximately fivefold higher than that in peripheral venous blood (79 μmol/L), suggesting significant hepatic metabolism of SCFAs (Cummings et al., 1987).

In porcine follicular fluid, the total SCFA concentration is approximately 150 μmol/L, composed of 86.91 μmol/L acetate, 8.65 μmol/L propionate, and 12.56 μmol/L butyrate (Xu et al., 2023). Currently, no data exist on the physiological concentrations of SCFAs in the testis or uterus.

## 3 Effects of short-chain fatty acids on intestinal barrier and intestinal peristalsis

### 3.1 Role of short-chain fatty acids in gut barrier

The gut barrier is the first line of defense of the gut and can prevent harmful substances in the gut from entering the body (Koh et al., 2016). Once the integrity of the gut barrier is compromised, it can cause bacterial infiltration and an unbalanced immune response, which are important mechanisms for the development of inflammatory bowel disease (IBD) (Dong et al., 2019). Short-chain fatty acids can maintain the integrity of the gut barrier by regulating the expression of tight junction proteins (Gonzalez et al., 2019; Yang et al., 2014; Mörkl et al., 2018). Among them, butyric acid is an important regulatory factor involved in the regulation of tight junction protein expression, and the regulatory effects of short-chain fatty acids such as acetic acid and propionic acid on tight junction protein expression are less studied (Ma et al., 2012; Feng et al., 2018). Butyrate can act as a stabilizer for hypoxia-inducible factor 1 (HIF-1), which is a transcription factor that coordinates barrier protection (Parada Venegas et al., 2019). HIF-1β deficiency can block the protective effect of butyrate on the gut barrier (Kelly et al., 2015). In rat intestinal epithelial cells, butyrate can promote the expression of CLDN1, increase transepithelial electrical resistance (TEER), and decrease paracellular flux (Wang et al., 2012). In the human colon cancer cell line Caco-2, butyrate maintains gut permeability by inhibiting the expression of permeation-promoting CLDN2 through an IL-10 Receptor Alpha-dependent mechanism (Zheng et al., 2017). Butyrate can promote the expression of mucin 2 (MUC2) and enhance the resistance of the mucosal layer to gut pathogens (Nielsen et al., 2018).

Another important mechanism involved in gut barrier function is the production of antimicrobial peptides by intestinal epithelial cells. Butyrate has been shown to induce gut cells to produce antimicrobial peptides involved in fighting pathogens (Zhao et al., 2018), and butyrate can increase TEER by activating Adenosine 5‘-monophosphate -activated protein kinase (Peng et al., 2009). Although positive effects of butyrate on the intestinal barrier have been noted in rodents and humans, conflicting data have been obtained in some *in vitro* studies. Vancamelbeke et al. demonstrated that treatment with butyrate (8 mM for 48 h) on human primary colon epithelial cells has a beneficial effect on TEER; however, in the same experiment, in the presence of inflammatory stimulants, butyrate could not improve the integrity of the epithelial barrier and even aggravated the destruction of its integrity (Vancamelbeke et al., 2019). At concentrations of 1–10 mM, butyrate significantly improved the epithelial barrier function of HT29-MTX-E12 human colon cells, while no beneficial effect was shown at 50–100 mM (Nielsen et al., 2018). These results are consistent with a study using human intestinal Caco-2 cells (Peng et al., 2007). Therefore, it is suggested that the effect of short-chain fatty acids on intestinal barrier function may be related to their concentration.

### 3.2 Effects of short-chain fatty acids on intestinal motility

**FIGURE 2:**
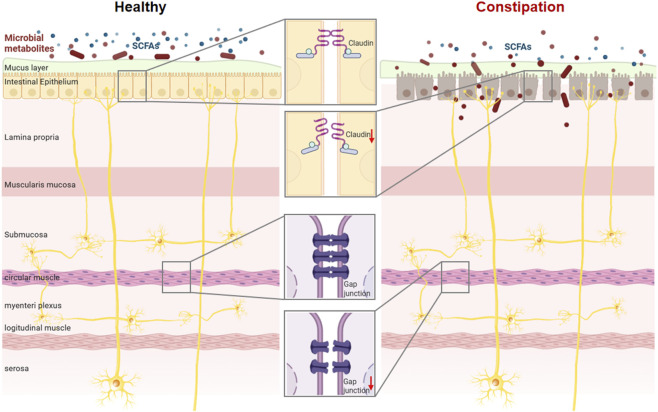
SCFAs are involved in maintaining intestinal barrier integrity and regulating intestinal motility. Dysbiosis of the gut microbiota impairs the synthesis of short-chain fatty acids and contributes to the pathogenesis of intestinal disorders.

The coordinated movement of ingested food to absorb nutrients and expel waste through the digestive tract (mainly including the gastrointestinal tract) is essential to the life of animals. In humans, the dynamic disturbance of the gut can exacerbate gut infections, cause poor absorption of nutrients, and lead to symptoms such as diarrhea or constipation. It has been recognized that gut microbiota disturbance can cause constipation(see Figure 2) (Wang et al., 2017). O-acetylated xylan from bamboo can relieve constipation by increasing short-chain fatty acids (Huang et al., 2022). In mice with dysbiosis, fecal microbiota transplantation also enhanced intestinal motility by increasing short-chain fatty acids in the gut (Jing et al., 2021). Some studies have shown that colon transport rates can be effectively increased by supplementing with butyrate (5 mM) (Shaidullov et al., 2021). In a rat model of IBS, total SCFAs enhanced proximal colonic contraction at 5–50 mM (Yuan et al., 2020).

To date, the molecular mechanisms of gut peristalsis have been extensively studied. It was recognized as early as 1899 that gut neurons located in the intestinal wall are organized into reflex pathways that directly or indirectly detect luminal stretching, regulate the activity of myogenic contractions, and drive directed propulsion of luminal contents (Bayliss and Starling, 1899). The role of gap junctions in intestinal peristalsis is critical, especially for the gap junctions composed of CX43 in Cajal interstitial cells and smooth muscle cells, which control intestinal peristalsis (Nemeth et al., 2000; Daniel and Wang, 1999). CX43 mediates calcium ion responses in mouse gut glial cells and regulates colonic transport (McClain et al., 2014). CX43 knockout in gut smooth muscle cells results in changes in gut motor response and muscle contractile force (Döring et al., 2007). Stapf aqueous extract ameliorates loperamide-induced constipation in mice by promoting CX43 expression (Gao et al., 2022). CX43 is also involved in maintaining the gut barrier; increased CX43 expression in gut epithelial cells enhances gut barrier function during acute and chronic inflammatory injury (Ey et al., 2009).

On the contrary, literature is scarce on the effect of SCFAs on the function of gap junction connexin in gut cells. However, in other tissues, propionic acid was shown to improve ventricular electrical remodeling in rats with myocardial infarction by regulating the expression and distribution of CX43 (Zhou et al., 2021). Sodium butyrate can enhance gap junctions between liver cells by promoting the expression of CX43 (Jung et al., 2006). These results indicate that short-chain fatty acids can affect the expression of CX43 and play a regulatory role in gap junctions.

## 4 The role of claudins and connexins in reproductive organs

### 4.1 Ovary

In the ovaries of female animals, by immunostaining with different molecular weights of serum proteins such as albumin, immunoglobulin (Ig) G1 heavy chain, inter-alpha-trypsin inhibitor, fibrinogen, and IgM reveals the presence of the blood-follicular barrier. Researchers have found that low molecular weight albumin is present in blood vessels, ovarian stroma, and developing follicles. However, IgG1 heavy chain and inter-alpha-trypsin inhibitor in follicles were significantly decreased, while IgM was significantly reduced in the entire interstitium outside the vessel, suggesting that the blood-follicle barrier may have different permeability to proteins of different molecular weights (Zhou et al., 2007). However, even in the presence of a blood-follicular barrier structure, follicular fluid is essentially similar to plasma (Angelucci et al., 2006). So, the structure of the blood-follicle barrier has received little attention from researchers. Currently, tight junction protein 1 is thought to be involved in the formation of the blood-follicular barrier in the ovary, but the claudin proteins are highly variable across species (Mora et al., 2012). Functional studies of claudins have focused on ovarian cancer, but the effect of claudins on follicular development is unknown. The results of single-cell transcriptome sequencing showed that the expression of CLDN3 in granulosa cells increased significantly with the increase of follicle diameter, and CLDN18 was abundant in oocytes (Zhang et al., 2018). Chang He et al. pointed out that CLDN4 is involved in the formation of follicles; knocking down CLDN4 in the ovaries *in vitro* resulted in blocked follicular formation and reduced expression of steroid hormone synthesis-related genes in the ovaries (Wang et al., 2023). Claudins are regulated by gonadotropins such as LH, FSH, HCG, and PMSG in the ovaries and show sensitivity to estrogen, but none of these articles reported the effects of claudins on ovarian function.

CX43 and CX37 are the main gap connexins in the ovary. CX37 and CX43 are essential for follicular growth. In the ovary, CX37 is located on the surface of oocytes, while CX43 is mainly located in granulosa cells, and the gap junctions formed by CX43 and CX37 establish metabolic coupling between oocytes and granulosa cells (Winterhager and Kidder, 2015). When the CX37 encoded by Gja4 gene is knocked out, oocytes lose the ability to undergo meiosis, stop growing, and eventually die due to metabolic decoupling from granulosa cells (Simon et al., 1997). Granulosa cells in follicles lacking CX43 are less responsive to oocyte paracrine factors, resulting in reduced granulosa cell proliferation (Tong et al., 2006). The expression and function of CX37 and CX43 are similar in different species. Gap junctions between follicles can also increase the concentration of cGMP in oocytes by transporting cGMP to inhibit the recovery of oocyte meiosis (Jaffe and Egbert, 2017). All these suggest that gap junctions play an important role in follicle development.

Short-chain fatty acids could be detected in follicular fluid. Naisheng Lu et al. reported that butyric acid promoted the secretion of progesterone and estradiol through the cAMP signaling pathway in pig granulosa cells (Lu et al., 2017). Qianhong Ye et al. reported that butyrate could promote the synthesis of estradiol and progesterone in rat primary ovarian granulosa cells and human granulosa KGN cells; additionally, butyrate can activate PGC1α to enhance mitochondrial dynamics and reduce oxidative damage (Ye et al., 2021). Kailu Liu et al. reported that butyric acid supplementation in obese mice could improve ovarian function and reduce the expression of local ovarian inflammatory factors (Liu et al., 2023). Butyrate can also be involved in the regulation of autophagy and apoptosis of Chinese hamster ovarian (CHO) cells (Lee and Lee, 2012). Although there have been many studies on sodium butyrate in the ovary, no studies have reported the effect of sodium butyrate on the expression of gap connexins and claudins in ovarian cells.

### 4.2 Uterus

In the uterus, the tight junction between endometrial epithelial cells is responsible for maintaining the compartments between the uterine cavity and endometrial tissue; tight junctions regulate the composition of the lumen fluid by limiting the passage of ions and molecules (Tsukita and Furuse, 2000). CLDN1, -3, -7, and -10 have been demonstrated in mouse endometrium (Wang et al., 2004; Schumann et al., 2015; Liang et al., 2013), and CLDN1, -3, -4, -5, and -7 are also present in rat and human endometrial epithelium (Nicholson et al., 2010; Poon et al., 2013; Mendoza-Rodríguez et al., 2005; Orchard and Murphy, 2002; Pan et al., 2009; Buck et al., 2012). In the non-receptive endometrium, the endometrial epithelium forms a complete polarized epithelial barrier (Ye, 2020). In the endometrium of non-pregnant mice, the CLDN3 protein is primarily located at the apex of lumen and glandular epithelial cells and shows additional localization in the basolateral membrane of the lumen epithelium (Schumann et al., 2015). After knocking out Cldn3 in endometrial epithelial cells, embryos could implant in the uterus, but a reduction in the number of implantation sites and litter size may suggest a higher incidence of implantation failure in Cldn3-KO mice (Grund et al., 2022).

Gap connexins are also associated with embryo implantation. In preparation for embryo implantation, the uterine epithelium differentiates into a receptive state within a short period, which is a prerequisite for allowing the blastocyst to adhere and invade. CX26 is mainly expressed in the endometrial epithelium of rodents; CX32 was also expressed in human and baboon endometrial epithelium (Winterhager et al., 1993; Jahn et al., 1995). In all reported species, CX43 is expressed only in endometrial stromal cells (Jahn et al., 1995; Winterhager et al., 2009). The expression of these connexins is regulated by estrogen and progesterone. With the increase of estrogen, the expression level of connexins increases significantly, and with the increase of progesterone, the expression level of connexins decreases significantly (Jahn et al., 1995). To explore the importance of CX26 for implantation, Diao et al. injected the non-specific gap junction blocker carbenoxolone (CBX) into mice prior to embryo attachment, and CBX disrupted the implantation process (Diao et al., 2013). For CX43, connexin 43 is considered to be a major gap connexin that helps synchronize contractile activity; progesterone inhibits uterine contractions by inhibiting the expression of CX43 in the uterus, thereby preventing premature delivery (Renthal et al., 2010).

**FIGURE 3:**
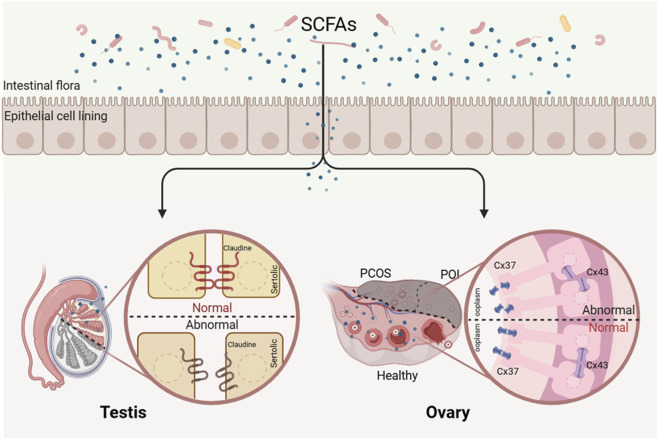
Deficiency of SCFAs leads to reproductive disorders. Short-chain fatty acids may regulate reproductive processes by modulating the expression of tight junction and gap junction proteins in reproductive organs.

Effects of short- and medium-chain fatty acids on lipid metabolism, pregnancy outcome, and embryo implantation have been reported. Short- or medium-chain fatty acids have the potential to prevent miscarriage in women or loss of early pregnancy in mammals (Ye et al., 2019). Sodium butyrate could improve the receptivity of porcine endometrial epithelial cells by enhancing acetylation of histone H3K9 (Ye et al., 2023), and butyrate alleviated lipopolysaccharide-induced endometritis in mice by inhibiting the inflammatory response (Guo et al., 2019). *In vitro*, sodium butyrate could enhance the differentiation of endometrial cells in Ishikawa (Fleming et al., 1995). *In vitro*, *Clostridium* butyricum significantly increased the expression of tight junction proteins claudin-3 and occludin to improve the endometrial barrier (see Figure 3). (Wang et al., 2022) This suggests that SCFA plays an important role in the uterus.

### 4.3 Testis

Claudins are quaternary transmembrane proteins encoded by the polygenic family CLDN and are present in all epithelial and endothelial cells. They are an important part of tight junctions, located in the apical membrane of epithelial cells, connecting the membranes of neighboring cells through self-aggregation and cell-to-cell interactions to form a barrier (Angelow et al., 2008). In the testis, claudins are of particular interest due to their involvement in the formation of the blood-testis barrier (BTB). The BTB is an important structure in the testis, which has a significant effect on the spermatogenesis of male animals. As early as the early 20th century, researchers found that when dyes are injected into adult rats, these dyes cannot penetrate the seminiferous tubules of adult rats, but they can penetrate those of juvenile rats, which led researchers to realize that after rats reach adulthood, a structure blocking dye penetration appears in the seminiferous tubules, known as the blood-testis barrier (Kormano, 1967; Korman and o, 1967). In mammals, the BTB is formed by specialized connections between adjacent Sertoli cells in the spermatogenic epithelium near the basement membrane. When the blood-testis barrier is destroyed, mice cannot complete the spermatogenesis process. Twenty-three different claudin proteins have been identified to date, and these different claudin proteins are considered to be seal- or barrier-specific; claudin-3, claudin-5, and claudin-11 are associated with blood-testis barrier formation (Günzel and Yu, 2013; Meng et al., 2005; Morrow et al., 2009; Gow et al., 1999). It is known that the testes of claudin-11-deficient mice are unable to establish a functional blood-testis barrier, and these mice are also infertile (Gow et al., 1999). There is clear evidence for the establishment and maintenance of the BTB by gut microbiota. A complete functional blood-testis barrier cannot be detected in the blood-brain barrier and the testis of germ-free mice (Braniste et al., 2014; Al-Asmakh et al., 2014), and the process of spermatogenesis was also seriously affected in germ-free mice, suggesting that the presence of gut microbiota is necessary for spermatogenesis (Al-Asmakh et al., 2014). In germ-free mice, researchers could rescue the blood-brain barrier by colonizing a single strain of bacteria that primarily produces butyrate or by administering sodium butyrate supplementation through oral force-feeding. However, there is still a lack of research on the effects of short-chain fatty acids on the BTB.

For gap junctions, subtypes such as CX26, CX32, CX33, CX46, and CX50 are found in the testes, but CX43 appears to be the most dominant gap junction in Sertoli cells and spermatogenic epithelial cells (Kidder and Cyr, 2016; P et al., 2015). Sertoli cells are considered to be the center of communication in the spermatogenic tubule. Gap junctions between Sertoli cells via CX43 form an intercellular communication network and indirectly synchronize germ cell proliferation and differentiation through metabolic and signaling coupling (Risley et al., 2002; Decrouy et al., 2004). CX43 is also thought to be essential for maintaining the homeostasis of the blood-testis barrier (Li et al., 2010). The testicular volume of CX43-specific knockout mice was significantly smaller than that of wild-type mice. Due to the absence of CX43, the Sertoli cells in the testis of CX43-KO adult male mice continued to proliferate, resulting in an increase in the number of Sertoli cells in the seminiferous tubule and a decrease in the number of spermatogonia (Brehm et al., 2007; Sridharan et al., 2007). Other gap junction proteins could not compensate for the role of CX43 in the testis (Carette et al., 2010). As mentioned earlier, the regulatory role of SCFAs on CX43 has been described in other tissues but remains unknown in the testis (see Figure 3).

## 5 Short-chain fatty acid imbalance and reproductive dysfunction

### 5.1 Role of short-chain fatty acids in polycystic ovary syndrome

PCOS is one of the most prevalent endocrine and metabolic disorders among women of reproductive age, with a global prevalence ranging from 4% to 21%. Its clinical manifestations are highly heterogeneous, typically characterized by hirsutism, oligomenorrhea or amenorrhea, chronic anovulatory infertility, obesity, and polycystic ovarian morphology on ultrasound (Stein and Leventhal, 1935). The pathogenesis of PCOS is complex and highly heterogeneous, with current evidence identifying obesity (particularly central obesity), insulin resistance, hyperandrogenemia, and chronic low-grade inflammation as its core pathophysiological foundations (Escobar-Mor and reale, 2018). In a study by our team, we found that the granulosa cells of PCOS patients were less resistant to oxidative stress (Ma et al., 2022).

Recent advances in gut microbiome research have revealed a close association between PCOS development and gut microbiota dysbiosis. Studies demonstrate that PCOS patients exhibit gut microbial ecological imbalance, marked by dual features of metabolic disturbances (Rodriguez Paris et al., 2022) and impaired intestinal barrier function (Liyanage et al., 2021). Specifically, the abundance of pro-inflammatory pathogens such as *Fusobacterium* and *Escherichia* is significantly elevated in PCOS patients. *Fusobacterium*, a conditional pathogen, exacerbates metabolic abnormalities by activating pro-inflammatory signaling pathways (e.g., NF-κB), increases intestinal permeability, and promotes systemic inflammation through endotoxin translocation (Gurung et al., 2020). *Escherichia* further disrupts gut barrier integrity by degrading tight junction proteins and enhancing mucosal invasion of commensal bacteria (Sartor, 2008). In PCOS patients, the expression of CX43 encoding gap Connexin was relatively low, and the oocyte maturation rate was significantly reduced (Liu et al., 2020). Moreover, the mutation of gap Connexin CX37 can cause the occurrence of PCOS in women (Guruvaiah et al., 2016). PCOS patients with higher expression of CX43 in granulosa cells had better pregnancy outcomes during IVF (Wang et al., 2009).And for women who undergo assisted reproductive treatment, the gut microbiota in their bodies also undergoes changes, resulting in a significant reduction in the synthesis of short-chain fatty acids (Wu et al., 2024).

Concurrently, PCOS patients show marked depletion of short-chain fatty acid (SCFA)-producing commensals, including Butyricimonas, Blautia, Coprococcus, and the “anti-inflammatory guardian” Faecalibacterium prausnitzii (see Figure 3) (Qiu et al., 2013; Zhang et al., 2019). This depletion results in globally reduced fecal SCFA levels (acetate: 24.59 ± 8.94; propionate: 13.93 ± 3.84; butyrate: 5.05 ± 1.59; valerate: 0.55 ± 0.29 μmol/g), significantly lower than healthy controls (acetate: 57.36 ± 9.33; propionate: 20.14 ± 5.96; butyrate: 12.86 ± 4.2; valerate: 1.66 ± 0.64 μmol/g) (Zhang et al., 2019). Butyrate deficiency is particularly critical: it regulates gene expression via histone deacetylase (HDAC) inhibition, promotes regulatory T cells (Tregs) differentiation, and maintains mucosal immune homeostasis (Zhang et al., 2025). Additionally, butyrate enhances gut barrier function and stimulates glucagon-like peptide-1 (GLP-1) secretion via FFAR2 activation, improving insulin sensitivity (Tolhurst et al., 2012).And for women who undergo assisted reproductive treatment, the gut microbiota in their bodies also undergoes changes, resulting in a significant reduction in the synthesis of SCFAs (Tolhurst et al., 2012). Animal studies confirm SCFAs’ protective role: colonization with Bifidobacterium lactis V9 elevates SCFA levels, reduces LH/FSH ratios, and ameliorates ovulatory dysfunction in PCOS models (Qiu et al., 2013). These findings underscore the pivotal role of the gut microbiota-SCFA axis in PCOS pathogenesis.

### 5.2 Role of short-chain fatty acids in premature ovarian insufficiency

Premature ovarian insufficiency (POI) is defined as ovarian functional failure in women under 40 years old due to depletion of ovarian reserve (Webber et al., 2017). Its clinical characteristics include abnormally elevated gonadotropin levels (e.g., follicle-stimulating hormone [FSH] often exceeding 25 IU/L), significantly reduced estrogen levels, and associated symptoms such as amenorrhea, infertility, and perimenopausal manifestations (e.g., hot flashes, osteoporosis), with a global prevalence of approximately 3.7% (Panay et al., 2020). The pathological phenotype of POI exhibits high severity and irreversibility, primarily attributed to genetic factors (e.g., X chromosome abnormalities, FMR1 gene premutation), autoimmune disorders (e.g., autoimmune oophoritis), iatrogenic damage (e.g., radiotherapy/chemotherapy or ovarian surgery), and specific infections (e.g., mumps virus), which collectively account for 70%–90% of POI cases (Panay et al., 2020). Although traditional views emphasize these direct damaging factors as core etiologies of POI, recent studies have gradually revealed potential connections between gut microbiota and ovarian aging. Current research has identified gut dysbiosis in POI patients accompanied by intestinal barrier impairment and bone loss (Zhang Y. W. et al., 2022). Notably, fecal transplantation has been shown to mitigate microbiota alterations in POI mouse models, increase short-chain fatty acid (SCFA) levels in feces, and reduce intestinal permeability (Huang et al., 2024).

### 5.3 Role of short-chain fatty acids in male reproductive health

Many factors can alter the composition of gut microbiota, thereby influencing the production of SCFAs in the gut. These factors include imbalanced diets (high-fat or restrictive), antibiotic misuse, and pesticide residues (Makki et al., 2018; Patangia et al., 2022; Ma et al., 2024). Particularly in modern society, high-fat diets and pesticide residues represent two major risk factors contributing to declining male sperm quality-a key driver of rising male infertility rates.For instance, high-fat diets suppress Bifidobacterium abundance, reduce gut SCFAs levels, and disrupt the blood-testis barrier (Li et al., 2019). Notably, fecal microbiota transplantation (FMT) has been shown to restore gut microenvironments and ameliorate high-fat diet (HFD)-induced spermatogenesis dysfunction (Ding et al., 2020; Hao et al., 2022; Zhang T. et al., 2022).Similarly, exposure to pesticide residue substances like trifloxystrobin led to a significant decrease in the relative abundance of the probiotic Parabacteroides; a significant reduction in the relative abundance of SCFAs in the gut was observed, along with a significant reduction in serum steroid hormones, with the structure of the seminiferous tubules being damaged and sperm quality significantly declining (see Figure 3) (Ma et al., 2024)

## 6 Summary

**FIGURE 4:**
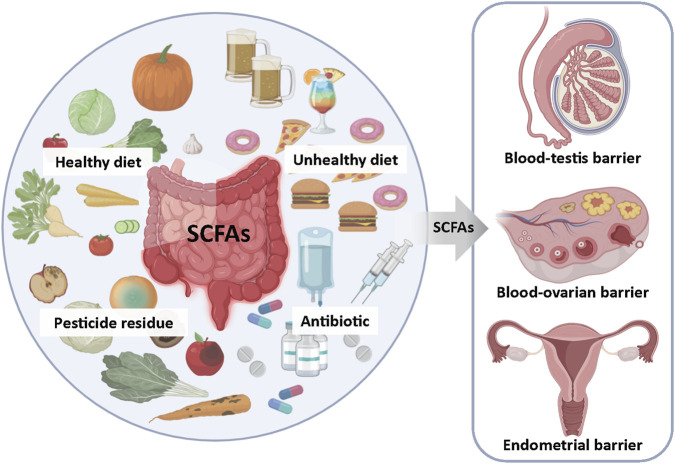
Gut Microbiota-Derived SCFAs: A Key Mediator of Environmental Stress Effects on Reproductive Health. Unbalanced diet (high-fat or restrictive diet), overuse of antibiotics and pesticide residues can alter the composition of intestinal microorganisms, affect the production of short-chain fatty acids (SCFAs), and thereby influence reproductive health.

It is well-established that the gut microbiota exerts broad and profound effects on human health, yet the mechanistic understanding of microbiota-derived metabolites in reproductive physiology remains incomplete. Unbalanced diets (such as high-fat diets or restrictive diets), excessive use of antibiotics, and pesticide residues all have an impact on reproductive health (see Figure 4).We hypothesize that microbiota-derived metabolites capable of modulating claudin and connexin expression may systemically influence these junctional complexes beyond the gut. Consequently, dysregulated claudin and connexin expression triggered by metabolite imbalances could serve as a unifying mechanism linking gut dysbiosis to pathologies in distal organs, including reproductive tissues. This review synthesizes current knowledge on the biosynthesis and tissue-specific distribution of short-chain fatty acids (SCFAs, prototypical microbiota metabolites), while systematically evaluating the functional roles of major claudins (e.g., CLDN3, CLDN11) and connexins (e.g., CX43, CX37) within testicular, ovarian, and uterine microenvironments. By exploring the crosstalk between gut microbiota metabolites and reproductive organs, this study encourages further investigation into their possible effects on gamete development and hormone secretion.
